# Molecular epidemiology of carbapenem-resistant gram-negative bacilli in Ecuador

**DOI:** 10.1186/s12879-024-09248-6

**Published:** 2024-04-06

**Authors:** Claudia Soria-Segarra, Carmen Soria-Segarra, Marcos Molina-Matute, Ivanna Agreda-Orellana, Tamara Núñez-Quezada, Kerly Cevallos-Apolo, Marcela Miranda-Ayala, Grace Salazar-Tamayo, Margarita Galarza-Herrera, Victor Vega-Hall, José E. Villacis, José Gutiérrez-Fernández

**Affiliations:** 1 Sosecali, Medical Services, Guayaquil, EC 090308 Ecuador; 2 Faculty of Medical Sciences, Guayaquil University, Guayaquil, Ecuador; 3 Department of Microbiology, School of Medicine and PhD Program in Clinical Medicine and Public Health, University of Granada & ibs, Granada, Spain; 4 Department of Internal Medicine, School of Medicine, Universidad Católica de Santiago de Guayaquil, Guayaquil, Ecuador; 5 Hospital del Río, Cuenca, Ecuador; 6 Hospital del Instituto Ecuatoriano de Seguridad Social Dr. Teodoro Maldonado Carbo, Guayaquil, Ecuador; 7 Hospital de Infectología Dr. José Daniel Rodríguez Maridueña, Guayaquil, Ecuador; 8 Omni Hospital, Guayaquil, Ecuador; 9 Hospital Vozandez, Quito, Ecuador; 10 Hospital Eugenio Espejo, Quito, Ecuador; 11 Interhospital, Guayaquil, Ecuador; 12 Centro de Investigación Para La Salud en América Latina (CISeAL), Pontificia Universidad Católica del Ecuador, Quito, 1701-2184 Ecuador; 13 Department of Microbiology, Hospital Virgen de Las Nieves, Institute for Biosanitary Research-Ibs, Granada, Spain

**Keywords:** Carbapenem resistance, Carbapenemase genes, Ecuador, Gram-negative bacilli

## Abstract

**Introduction:**

Carbapenem-resistant gram-negative bacilli are a worldwide concern because of high morbidity and mortality rates. Additionally, the increasing prevalence of these bacteria is dangerous. To investigate the extent of antimicrobial resistance and prioritize the utility of novel drugs, we evaluated the molecular characteristics and antimicrobial susceptibility profiles of carbapenem-resistant Enterobacterales, *Pseudomonas aeruginosa* and *Acinetobacter baumannii* in Ecuador in 2022.

**Methods:**

Ninety-five clinical isolates of carbapenem non-susceptible gram-negative bacilli were collected from six hospitals in Ecuador. Carbapenem resistance was confirmed with meropenem disk diffusion assays following Clinical Laboratory Standard Institute guidelines. Carbapenemase production was tested using a modified carbapenemase inactivation method. Antimicrobial susceptibility was tested with a disk diffusion assay, the Vitek 2 System, and gradient diffusion strips. Broth microdilution assays were used to assess colistin susceptibility. All the isolates were screened for the *bla*_KPC,_ *bla*_NDM,_ *bla*_OXA-48,_ *bla*_VIM_ and *bla*_IMP_ genes_._ In addition, *A. baumannii* isolates were screened for the *bla*_OXA-23_, *bla*_*OXA*-58_ and *bla*_OXA-24/40_ genes.

**Results:**

Carbapenemase production was observed in 96.84% of the isolates. The *bla*_KPC_, *bla*_NDM_ and *bla*_OXA-48_ genes were detected in Enterobacterales, with *bla*_KPC_ being predominant. The *bla*_VIM_ gene was detected in *P. aeruginosa,* and *bla*_OXA-24/40_ predominated in *A. baumannii*. Most of the isolates showed co-resistance to aminoglycosides, fluoroquinolones, and trimethoprim/sulfamethoxazole. Both ceftazidime/avibactam and meropenem/vaborbactam were active against carbapenem-resistant gram-negative bacilli that produce serin-carbapenemases.

**Conclusion:**

The epidemiology of carbapenem resistance in Ecuador is dominated by carbapenemase-producing *K. pneumoniae* harbouring *bla*_KPC_. Extensively drug resistant (XDR) *P. aeruginosa* and *A. baumannii* were identified, and their identification revealed the urgent need to implement strategies to reduce the dissemination of these strains.

**Supplementary Information:**

The online version contains supplementary material available at 10.1186/s12879-024-09248-6.

## Introduction

The increasing number of multidrug-resistant organisms (MDROs) constitutes a major threat to health worldwide. MDROs are linked to increasing costs and mortality rates [1] and are an important challenge to clinicians due to the complicated choice of treatment option [2].

The World Health Organization included carbapenem-resistant *Acinetobacter baumannii, Pseudomonas aeruginosa*, and Enterobacterales as “priority antibiotic-resistant pathogens”, for which the research and development of new antimicrobial agents is critical [3]. The dissemination of these pathogens is mainly due to high-risk clones and is generally associated with health care-associated infections with high mortality rates [4, 5].

The mechanisms of carbapenem resistance are diverse but are dominated by the expression of carbapenemases. The prevalence of carbapenemases varies according to region [2]. In Ecuador, despite the high prevalence of carbapenem-resistant Enterobacterales (CRE) (37%) and other gram-negative rod-shaped bacteria [6, 7], there is little information about the mechanisms involved and the antimicrobial susceptibility profiles, including that to recently approved antibiotics such as ceftazidime/avibactam, meropenem/vaborbactam, or ceftolozane/tazobactam, which constitute the first line of treatment against carbapenem-resistant gram-negative rod-shaped bacteria [8, 9].

Thus, essential information is required to optimize the administration of antibiotics as a lack of knowledge could lead to the misuse of antibiotics and the rapid development of antimicrobial resistance.

Therefore, to clarify the extent of antimicrobial resistance and prioritize the utility of newly available drugs, our goal was to determine the molecular characteristics and antimicrobial susceptibility profiles of clinical CRE, *P. aeruginosa* and *A. baumannii* complex isolates from Ecuador.

## Methodology

A multicentre study was carried out in six hospitals in Ecuador between January 2022 and May 2022. Clinical isolates of CRE, *P. aeruginosa* and the *A. baumannii* complex were collected according to protocols established by each institution. Carbapenem resistance was defined when the isolate was non-susceptible to any of the carbapenems tested according to Clinical Standards Institute (CLSI) breakpoints [10]. Only one clinical isolate from each patient was studied, and there was a preference for samples from sterile sites or those with the greatest resistance phenotype.

CRE isolates were cultured on CHROMagar Super Carba (CHROMagar, France). *P. aeruginosa* and *A. baumannii* complex isolates were cultured on MacConkey agar (16–18 h; 35 °C) (Becton–Dickinson, England) to check their viability and purity. Isolates were identified with the Vitek 2 System (BioMérieux, France) and conventional biochemical tests.

Carbapenem resistance was confirmed with meropenem disk diffusion assays using CLSI methodology. Carbapenemase production was studied with the modified carbapenem inactivation method (mCIM) for Enterobacterales and *P. aeruginosa* according to CLSI guidelines [10]. *A. baumannii* complex isolates were studied with the optimized carbapenem inactivation method described by Zhang S. et al. [11].

### Antimicrobial susceptibility testing

Susceptibility tests for ciprofloxacin, amikacin, gentamicin, trimethoprim/sulfamethoxazole, tigecycline, ceftazidime, cefepime, meropenem imipenem, ampicillin/sulbactam and piperacillin/tazobactam were performed using disk diffusion assays and the Vitek 2 System (AST-N402 or AST-N401 Card) (BioMérieux, France). Minimal inhibitory concentrations (MICs) for ceftazidime/avibactam and meropenem/vaborbactam were determined using gradient diffusion strips (Liofilchem, Italy). MIC values and disk diffusion results were interpreted using CLSI breakpoints [10]. For tigecycline, the U.S. Food and Drug Administration (FDA) breakpoints were used (susceptible ≥ 19 mm or ≤ 2 µg/ml) for Enterobacterales [12]. For meropenem/vaborvactam, European Committee on Antimicrobial Susceptibility (EUCAST) testing breakpoints were used for *P. aeruginosa* (susceptible ≤ 8 µg/ml) [13].

Susceptibility to ceftazidime/avibactam and meropenem/vaborbactam was not tested in NDM-positive isolates.

The MIC of colistin was obtained using a broth microdilution (CBM) method, as described in CLSI document M07-A8 [14]. Analytical grade colistin sulfate (Sigma‒Aldrich Code C2700000, batch 3.0) and Mueller Hinton broth with cation adjustment (Thermo Fischer Scientific, United Kingdom) were used. The concentration range was 0.5–4 µg/mL, and CLSI breakpoints were used to define colistin resistance (MIC values ≥ 4 µg/mL) [10]. The MIC_50_ and MIC_90_ were determined for each antimicrobial.

*Escherichia coli* ATCC 25922, *P. aeruginosa* ATCC 27853, *K. pneumoniae* BAA ATCC 1705 and *E. coli* AR Bank #0349 were used for quality control.

### Detection of carbapenemase and mcr-1 genes

The carbapenemase-encoding genes *bla*_KPC,_ *bla*_NDM,_ *bla*_OXA-48,_ *bla*_VIM,_ and *bla*_IMP_ were studied using multiplex polymerase chain reaction (PCR) in all isolates [15]. Additionally, the *bla*_OXA-23-like_, *bla*_*OXA*-58_ and *bla*_OXA-24/40-like_ genes were studied in *A. baumannii* complex isolates with a previously described multiplex PCR method [16].

The molecular detection of the colistin-resistance gene *mcr-1* was performed via PCR [17].

### Clonality study

Clonal relatedness was studied in *bla*_KPC_-positive *K. pneumoniae, bla*_VIM-_positive *P. aeruginosa* and *bla*_OXA-24/40-_positive *A. baumannii* complex using ERIC-PCR following a previously described method [18].

Dendrograms were constructed using GelJ software based on Dice’s similarity coefficient and the unweighted pair group method (UPGM) (tolerance 2%). The cut-off level for ERIC-PCR electrophoretic pattern delineation was 80% similarity.

## Results

One hundred and twenty-nine isolates were obtained for analysis. Thirty-four were rejected for inconsistencies in shipments or duplications (26.35%). Thus, a total of ninety-five isolates were included in the study (73.64%).

Enterobacterales predominated among the sample and accounted for 60 isolates studied (63.15%), with *K. pneumoniae* being the most frequently isolated species (*n* = 45; 47.36%). *K. aerogenes, E. cloacae* and *E. coli* were also present in 16.66% (*n* = 10), 5% (*n* = 3) and 3.33% (*n* = 2) of samples, respectively. The *A. baumannii* complex was present in 25.26% (*n* = 24) of the samples, and *P. aeruginosa* was present in 11.57% (*n* = 11) of the samples.

Carbapenemase production was confirmed in 92 isolates (96.84%). Three *P. aeruginosa* isolates tested negative for mCIM.

*Bla*_KPC_ was the most frequent carbapenemase-encoding gene found in Enterobacterales (*n* = 52; 86.66%). *Bla*_NDM_ (*n* = 7; 11.66%) and *bla*_OXA-48_ (*n* = 1; 1.66%) were also detected. Neither *bla*_IMP_ nor *bla*_VIM_ was detected in Enterobacterales. Both ceftazidime/avibactam (MIC_50_ and MIC_90_ <  = 0.12/4 µg/ml) and meropenem/vaborbactam (MIC_50_ 0.064/8 µg/ml; MIC_90_ 0.84/8 µg/ml) were active against all tested isolates harbouring *bla*_*KPC*_. The susceptibility rates to CPE were 95% and 76.67% for tigecycline and colistin, respectively.

Detailed information about the susceptibility patterns of the CPE strains, *K. pneumoniae* strains *and K. aerogenes bla*_KPC_ strains is provided in Table 1.

**Table 1:** Antimicrobial susceptibility against carbapenemase-producing Enterobacterales

Antimicrobials	CPE (*n* = 60)		*K. pneumoniae bla*_KPC_ (*n* = 40)				*K. pneumoniae bla*_NDM_ (*n* = 5)				*Klebsiella aerogenes bla*_KPC_ (*n* = 9)			
	Susceptible		Susceptible		MIC_50_ *µ*g/ml	MIC_90_ *µ*g/ml	Susceptible		MIC_50_ *µ*g/ml	MIC_90_ *µ*g/ml	Susceptible		MIC_50_ *µ*g/ml	MIC_90_ *µ*g/ml
	No	%	No	%			No	%			No	%		
**AK**	27	45.00	21	47.73	4	≥ 64	0	0.00	≥ 64	≥ 64	2	22.22	≥ 64	≥ 64
**GN**	11	18.33	8	18.18	≥ 64	≥ 64	0	0.00	≥ 64	≥ 64	2	22.22	≥ 64	≥ 64
**CIP**	8	13.33	5	11.36	≥ 4	≥ 4	1	20.00	≥ 4	≥ 4	2	22.22	≥ 4	≥ 4
**SXT**	4	6.67	4	9.09	4/76	4/76	0	0.00	4/76	4/76	0	0.00	4/76	4/76
**TIG**	57	95.00	38	86.36	≤ 0,5	≤ 0,5	5	100.00	2	2	9	100.00	≤ 0.5	≤ 0.5
**COL**	46	76.67	34	85.00	1	≥ 8	5	100.00	1	1	1	11.11	≥ 8	≥ 8
**CAZ/AVI**	53^a^	100.00	40	100.00	≤ 0.12/4	≤ 0.12/4	NT	NT	NT	NT	9	100.00	DD	DD
**MER/VAB**	25^b^	100.00	23^c^	100.00	0.064/8	0.94/8	NT	NT	NT	NT	1^d^	100.00	-	-
**CAZ**	0	0.00	0	0.00	≥ 64	≥ 64	0	0	≥ 64	≥ 64	0	0.00	≥ 64	≥ 64
**FEP**	1	0.00	0	0.00	≥ 32	≥ 32	0	0	≥ 32	≥ 32	0	0.00	≥ 32	≥ 32
**IMP**	1	3.33	0	0.00	≥ 16	≥ 16	0	0	≥ 16	≥ 16	0	0.00	≥ 16	≥ 16
**MER**	1	3.33	0	0.00	≥ 16	≥ 16	0	0	≥ 16	≥ 16	0	0.00	≥ 16	≥ 16
**PTZ**	0	0.00	0	0	≥ 128/4	≥ 128/4	0	0	≥ 128/4	≥ 128/4	0	0.00	≥ 128/4	≥ 128/4

*NT* not tested, *DD* disk diffusion, *AK* Amikacin, *GN* Gentamicin, *CIP* Ciprofloxacin, *SXT* Trimethoprim/sulfamethoxazole, *TIG* Tigecycline, *COL* Colistin, *CAZ/AVI* Ceftazidime/Avibactam, *MER/VAB* Meropenem/vaborbactam, *CAZ* Ceftazidime, *FEP* Cefepime, *IMP* Imipenem, *MER* Meropenem, *PTZ* Piperacillin/tazobactam

^a^53 isolates tested

^b^25 isolates tested

^c^23 isolates tested

^d^1 isolate tested

Tigecycline and colistin were the only drugs that *K. aerogenes bla*_*N*DM_ isolates were susceptible to (*n* = 1). *E. cloacae bla*_KPC_ isolates (*n* = 2) were resistant to most of the antimicrobials tested, except for amikacin tigecycline, colistin, ceftazidime/avibactam and meropenem/vaborbactam, as observed for *E. coli bla*_KPC_ (*n* = 1).

*E. cloacae bla*_NDM_ (*n* = 1) was only susceptible to colistin and amikacin.

The susceptibility profile of *E. coli bla*_oxa-48_ was the most conserved. This isolate was resistant to only ciprofloxacin, trimethoprim/sulfamethoxazole, and ceftazidime. Tigecycline, colistin, ceftazidime/avibactam, trimethoprim/sulfamethoxazole and colistin were active against *E. coli bla*_KPC._ These strains were resistant to ciprofloxacin, aminoglycosides and all beta-lactam antimicrobial agents tested.

In *P. aeruginosa,* only *bla*_VIM_ was detected (*n* = 8; 72.72%*).* Three isolates lacked any of the examined carbapenemase genes and tested negative for mCIM. All antimicrobials tested showed high resistance in *bla*_VIM_-positive *P. aeruginosa*. Ceftolozane/tazobactam was active against carbapenemase-negative *P. aeruginosa*.

In *A. baumannii* complex isolates, 66.66% (*n* = 16) of the patients were *bla*_OXA-24/40_ positive, and 33.33% (*n* = 8) of the patients were *bla*_OXA23_ positive. *bla*_KPC,_ *bla*_NDM,_ *bla*_OXA-48,_ *bla*_VIM,_ and *bla*_IMP_ were not detected in any of the isolates. *A. baumannii* complex isolates were only susceptible to colistin (95%), regardless of which gene was present in the isolates.

Tables 2 and 3 provide comprehensive details regarding carbapenem-resistant non-fermentative gram-negative pathogens. The MIC values of the antimicrobials evaluated are detailed in Supplementary Table 1.

**Table 2:** Susceptibility antimicrobial against *P. aeruginosa*

	*P aeruginosa bla*_VIM_ *n* = 8				*P.aeruginosa* mCIM negative *n* = 3				*P. aeruginosa n* = 11	
Antimicrobials	Susceptible		MIC_50_ *µ*g/ml	MIC_90_ *µ*g/ml	Susceptible		MIC_50_ *µ*g/ml	MIC_90_ *µ*g/ml	Susceptible	
	No	%			No	%			No	%
**AK**	4	50	4	32	2	66.67	16	16	6	54.54
**GN**	2	25	≥ 32	≥ 32	1	33.33	4	8	3	27.27
**CIP**	1	12.5	≥ 4	≥ 4	1	33.33	0.5	≥ 4	1	9.09
**COL**	4	50	2	≥ 8	3	100.00	1	1	7	63.63
**CAZ/AVI**	NT	NT	NT	NT	3^a^	100.00	DD	DD	3^a^	100
**MER/VAR**	NT	NT	NT	NT	3^a^	100.00	0.5	1	3^a^	100
**CAZ**	0	0	≥ 64	≥ 64	2	66.67	4	≥ 64	2	18.18
**FEP**	0	0	≥ 32	≥ 32	1	33.33	8	≥ 32	1	9.09
**IMP**	0	0	≥ 16	≥ 16	0	0.00	≥ 16	≥ 16	0	0
**MER**	0	0	≥ 16	≥ 16	0	0.00	≥ 16	≥ 16	0	0
**CEF/TAZ**	0	0	> 256	> 256	3	100.00	1.5	4	3	27.27
**PTZ**	0	0	≥ 128/4	≥ 128/4	0	0.00	≥ 128/4	≥ 128/4	0	0

*NT* not tested, *DD* Disk diffusion, *AK* Amikacin, *GN* Gentamicin, *CIP* Ciprofloxacin, *COL* Colistin, *CAZ/AVI* Ceftazidime/avibactam, *MER/VAB* Meropenem/vaborbactam, *CAZ* Ceftazidime, *FEP* Cefepime, *IMP* Imipenem, *MER* Meropenem, CEF/TAZ, *PTZ* Piperacillin/tazobactam

^a^EUCAST Breakpoints

**Table 3:** Susceptibility antimicrobial against *A. baumannii* complex

*A.baumannii* complex *bla*_OXA23_ *n* = 8					*A.baumannii* complex *bla*_OXA24/40_ *n* = 16				*A. baumannii* complex *n* = 24	
	Susceptible				Susceptible				Susceptible	
Antimicrobials	No	%	MIC_50_ *µ*g/ml	MIC_90_ *µ*g/ml	No	%	MIC_50_ *µ*g/ml	MIC_90_ *µ*g/ml	No	%
**AK**	0	0.00	≥ 64	≥ 64	0	0.00	≥ 64	≥ 64	0	0
**GN**	0	0.00	≥ 64	≥ 64	0	0.00	≥ 64	≥ 64	0	0
**CIP**	0	0.00	≥ 4	≥ 4	0	0.00	≥ 4	≥ 4	0	0
**SXT**	0	0.00	4/76	4/76	0	0.00	4/76	4/76	0	0
**COL**	6^a^	100.00	1	1	13^b^	92.86	1	1	19 ±	95
**CAZ**	2	25.00	≥ 64	≥ 64	2	12.50	≥ 64	≥ 64	4	18.18
**FEP**	0	0.00	≥ 32	≥ 32	0	0.00	16	≥ 32	0	0
**IMP**	0	0.00	≥ 16	≥ 16	0	0.00	≥ 16	≥ 16	0	0
**MERO**	0	0.00	≥ 16	≥ 16	0	0.00	≥ 16	≥ 16	0	0
**PTZ**	0	0.00	≥ 128/4	≥ 128/4	0	0.00	≥ 128/4	≥ 128/4	0	0
**SAM**	0	0.00	≥ 32/16	≥ 32/16	2	12.50	≥ 32/16	≥ 32/16	2	8.33

*AK* Amikacin, *GN* Gentamicin, *CIP* Ciprofloxacin, *SXT* Trimethoprim/sulfamethoxazole, *COL* Colistin, *CAZ* Ceftazidime, *FEP* Cefepime, *IMP* Imipenem, *MER* Meropenem, *PTZ* Piperacillin/tazobactam, *SAM* Ampicillin/sulbactam

^a^6 isolates tested

^b^14 isolates tested ± 20 isolates tested

None of the colistin-resistant isolates carried the *mcr-1* gene.

*Bla*_VIM-_positive *P. aeruginosa* and *bla*_KPC-_positive *K. pneumoniae* (*n* = 35) isolates exhibited significant genetic heterogenicity (Supplementary Figs. 1 and 2).

In the *bla*_OXA-24/40-_positive *A. baumannii* complex, six electrophoretic patterns were found, and 50% of the isolates belonged to only one pattern, indicating the clonal dissemination of this microorganism (Supplementary Fig. 3).

Four electrophoretic patterns were found in *bla*_KPC-_positive *K. aerogenes* (8 isolates studied) (Supplementary Fig. 4).

## Discussion

Our research describes the molecular characteristics of carbapenem-resistant gram-negative rod-shaped bacteria in Ecuador. Carbapenemase production was prevalent in the isolates studied (96.64%), and carbapenemase production has been described by several authors as the main mechanism involved in carbapenem resistance [6, 19–21].

Carbapenemase-encoding genes such as *bla*_KPC_, *bla*_NDM_ and *bla*_OXA-48_ were detected in Enterobacterales, and *bla*_KPC_ was the predominant gene. Our results are similar to those described in other Western countries, such as the United States, Argentina, Colombia, and Brazil, which described *bla*_KPC_ as the prevalent gene linked to *K. pneumoniae* [7, 21, 22]. Our results also agree with previous data published in 2022 by the National Reference Laboratory, which described *K. pneumoniae* harbouring *bla*_KPC_ as the main cause of carbapenem resistance in Enterobacterales in Ecuador [23].

In our study, we did not find *bla*_NDM_ (11.47%) or *bla*_OXA-48_ (1.64%) genes very frequently in Enterobacterales; these percentages are comparable to those reported in a national surveillance report [23]. However, given the small number of isolates, we advise using caution when considering this information. *Bla*_NDM_ was detected in *K. pneumoniae, K. aerogenes* and *E. cloacae.* Interestingly, we did not find any *P. rettgeri* isolates harbouring this gene because this species has been shown to play a central role in the dissemination of *bla*_NDM_ in Latin America, but infections by *P. rettgeri* are not very common [24]. Similar to other studies, we found *bla*_OXA-48_ only in *E. coli*_,_ which is the main species associated with this gene [25, 26].

The pattern observed in Ecuador is different from that described in other countries, such as India, where *NDM* is prevalent, or Turkey, where *OXA-48* dominates among CRE [26, 27].

During the COVID-19 pandemic, an increase in carbapenemase-producing Enterobacterales was observed; moreover, the emergence of isolates harbouring more than one carbapenemase gene was reported [28]. In 2021, Ecuador reported *bla*_KPC_ + *bla*_NDM_ and *bla*_KPC_ + *bla*_OXA-48_ associations [28, 29]. Nevertheless, we did not find any combinations of carbapenemase genes in any of the isolates studied.

Co-resistance to aminoglycosides and fluoroquinolones was observed in *K. pneumoniae bla*_KPC-_positive isolates. This association has also been described by several authors and is linked to horizontal dissemination [30, 31]. Genetic diversity was revealed in our isolates, suggesting horizontal dissemination. Interestingly, our results differ from those of previous research in which clonal circulation of *K. pneumoniae bla*_KPC-_positive isolates was documented [18]. The differences found could be attributed to the fact that our study was not limited to one city or hospital area, such as intensive care units, where most other research is focused.

According to our results, tigecycline could be considered a therapeutic alternative for Enterobacterales isolates, independent of carbapenemase genes or the species involved, but its use will require a previous antimicrobial susceptibility report, as other authors have also suggested [32].

Enterobacterales harbouring *bla*_KPC_ showed reduced susceptibility to colistin, but surprisingly, all isolates harbouring *bla*_NDM_ remained susceptible to colistin, contrary to the findings of other authors, who described an increase in colistin resistance in CRE isolates harbouring *bla*_NDM_ [33]_._ Based on our findings, colistin may be considered a significant therapeutic option for the treatment of CRE, but a prior susceptibility report may be needed. The *mcr*-1 gene was not detected in any of our isolates, in contrast with the results of other Latin American studies [34–36], suggesting that resistance could be mediated by chromosomal mutations or other *mcr* variants that were not studied and have been described in the region, including *mcr-3* or *mcr-5* [37–39]. Further studies are needed to understand the resistance mechanisms involved in colistin resistance.

New antimicrobial agents, such as ceftazidime/avibactam and meropenem/vaborbactam, demonstrated complete susceptibility in all the *bla*_KPC-_positive isolates. Despite the susceptibility patterns reported by several authors, the emergence of KPC isolates with ceftazidime/avibactam resistance has been reported, which has been frequently associated with mutations leading to substitutions in the Ω-loop of the KPC-3 variant [40, 41]. Fortunately, our research did not show any resistance to this new antimicrobial in KPC isolates, perhaps due to the recent introduction of this antimicrobial (June 2022) in Ecuador, although some cases of CAZ/AVI resistance have been reported prior to drug introduction in other countries through a salt bridge between glutamic 197 and arginine 164 in the wild-type KPC enzyme; however, this result was not found in this study [42].

Carbapenemase production by the *bla*_VIM_ gene was detected in 63.63% of *P. aeruginosa* isolates, and this gene encodes a metallo-β-lactamase. Our findings are consistent with several other authors who determined that this gene was prevalent [43]. *P. aeruginosa* exhibited high rates of co-resistance to aminoglycosides, ciprofloxacin, colistin and ceftolozane/tazobactam, rendering these antimicrobials ineffective for empirical treatment. Our findings differ from those of other authors who recommended ceftolozane/tazobactam as an effective treatment for multidrug-resistant *P. aeruginosa* [44]. In contrast to our findings, *Ajila* et al. reported good susceptibility to ceftolozane/tazobactam in *P. aeruginosa* isolates recovered in 2019 in Ecuador [45]. We attributed these conflicting findings to the molecular mechanisms involved in the studied isolates, but these hypotheses were not described by the authors.

In America, carbapenem resistance in *A. baumannii* complex isolates is principally mediated by oxacillinases, particularly *bla*_OXA-23_ and *bla*_OXA-58_ [46, 47]. In 2016, Nuñez-Quezada et al. reported an outbreak of a carbapenem-resistant *A. baumannii* complex with *bla*_OXA-72,_ a member of the *bla*_OXA-24/40_ subgroup, in Guayaquil, Ecuador [48]. Our research revealed that *bla*_OXA-24/40_ (66.66%) was predominant in *A. baumannii* complex isolates, similar to the results of a previous report in our country; furthermore, the same PCR pattern was observed in 50% of the isolates, which indicates the clonal spread of this microorganism, as has been previously reported [48].

Nevertheless, our results differ from those published in a national surveillance report, which showed a predominance of *OXA-23* in *A. baumannii* complex isolates collected from 2019 to 2021. However, an increase in *OXA-24/40* was observed in 2021, with similar values to those of *OXA-23* (*bla*_OXA-23_ *n* = 129; *bla*_OXA-24/40_ *n* = 116). In addition, we did not detect *bla*_OXA-58_ in our isolates*,* although it has been reported previously in Ecuador [23]. Our results also differ from the regional epidemiology [49], where the *bla*_OXA-24/40_ gene has been described only sporadically and is mainly reported in countries such as Taiwan, China and South Korea in Asia [50, 51].

Co-resistance to other groups of drugs is highly common in *A. baumannii* complex isolates, and this co-resistance reduces the number of therapeutic options. The extensive drug resistance (XDR) phenotype was independent of the oxacillinase gene. The isolates only showed 100% susceptibility to colistin, and colistin could be considered a last resort option for these XDR isolates.

This study has several limitations. First, our results could not be generalized to primary care hospitals or other cities as the hospitals were not randomly selected; instead, they included hospitals from public and private services from the second and third levels of attention in Ecuador´s most populous cities. Second, clinical isolates of carbapenem-resistant gram-negative bacilli were selected by each microbiology laboratory according to their protocols and clinical requests for culture by physicians; therefore, some isolates were not included. Third, the sensitivity and specificity of the mCIM method are greater than 90%, although there could be false-negative results due to uncommon carbapenemase types that were not tested in our study [52]. Finally, *bla*_OXA143_, which is an oxacillinase previously described in *A. baumannii* in Ecuador, has not been examined.

In conclusion, the epidemiology of carbapenem resistance in the three most important cities of Ecuador (Quito, Guayaquil, and Cuenca) was dominated by carbapenemase-producing *K. pneumoniae* harbouring *bla*_KPC_. Additionally, XDR *P. aeruginosa* and *A. baumannii* complex isolates were also present, showing an urgent need to implement strategies to reduce their dissemination.

### Supplementary Information

**Supplementary Material 1.**

**Supplementary Material 2.**

**Supplementary Material 3.**

**Supplementary Material 4.**

**Supplementary Material 5.**

## Data Availability

The datasets used during the current study are available from the corresponding author upon reasonable request.

## References

[1] Knols BG, Smallegange RC, Tacconelli E, Magrini N, Kahlmeter G, Singh N (2009). Global priority list of antibiotic-resistant bacteria to guide research, discovery, and development of new antibiotics. Lancet Infect Dis.

[2] Pournaras S, Zarrilli R, Higgins PG, Tsioutis C (2021). Editorial: carbapenemase-producing organisms as leading cause of hospital infections. Front Med.

[3] World Health Organization. WHO publishes list of bacteria for which new antibiotics are urgently needed. Available from: https://www.who.int/news/item/27-02-2017-who-publishes-list-of-bacteria-for-which-new-antibiotics-are-urgently-needed. [cited 2023 Aug 21].

[4] Kelesidis T, Karageorgopoulos DE, Kelesidis I, Falagas ME (2008). Tigecycline for the treatment of multidrug-resistant Enterobacteriaceae: a systematic review of the evidence from microbiological and clinical studies. J Antimicrob Chemother.

[5] Ko KS (2019). Antibiotic-resistant clones in Gram-negative pathogens: presence of global clones in Korea. J Microbiol.

[6] Sánchez-López J, Cantón R (2019). Current status of ESKAPE microorganisms in Spain: epidemiology and resistance phenotypes. Rev Espanola Quimioter Publicacion Of Soc Espanola Quimioter.

[7] García-Betancur JC, Appel TM, Esparza G, Gales AC, Levy-Hara G, Cornistein W (2021). Update on the epidemiology of carbapenemases in Latin America and the Caribbean. Expert Rev Anti Infect Ther.

[8] Tamma PD, Hsu AJ (2019). Defining the role of novel β-lactam agents that target carbapenem-resistant gram-negative organisms. J Pediatr Infect Dis Soc.

[9] Doi Y (2019). Treatment options for Carbapenem-resistant gram-negative bacterial infections. Clin Infect Dis.

[10] Clinical and Laboratory Standards Institute (2021). Performance Standards for Antimicrobial Susceptibility Testing; 31th informational supplement. CLSI document M100-S31.

[11] Zhang S, Mi P, Wang J, Li P, Luo K, Liu S (2023). The optimized carbapenem inactivation method for objective and accurate detection of carbapenemase-producing Acinetobacter baumannii. Front Microbiol.

[12] Food and Drug Aministration C for DE and FDA. FDA. Tigecycline – Injection products. 2023. Available from: https://www.fda.gov/drugs/development-resources/tigecycline-injection-products. [cited 2024 Jan 30].

[13] European Committee on Anitmicrobial Susceptibility Testing. Breakpoint tables for interpretation of MICs and zone diameters. Version 14.0, valid from 2024–01–01. Available from: https://www.eucast.org/fileadmin/src/media/PDFs/EUCAST_files/Breakpoint_tables/v_14.0_Breakpoint_Tables.pdf. [cited 2024 Jan 30].

[14] Clinical and Laboratory Standards Institute. Methods for dilution antimicrobial susceptibility tests for bacteria that grow aerobically; approved standard — Ninth Edition. Vol. 32, methods for dilution antimicrobial susceptibility tests for bacteria that grow aerobically; approved standard- Ninth Edition. 2012. p. 18.

[15] Poirel L, Walsh TR, Cuvillier V, Nordmann P (2011). Multiplex PCR for detection of acquired carbapenemase genes. Diagn Microbiol Infect Dis.

[16] Woodford N, Ellington MJ, Coelho JM, Turton JF, Ward ME, Brown S (2006). Multiplex PCR for genes encoding prevalent OXA carbapenemases in Acinetobacter spp. Int J Antimicrob Agents.

[17] Rebelo AR, Bortolaia V, Kjeldgaard JS, Pedersen SK, Leekitcharoenphon P, Hansen IM (2018). Multiplex PCR for detection of plasmid-mediated colistin resistance determinants, mcr-1, mcr-2, mcr-3, mcr-4 and mcr-5 for surveillance purposes. Eurosurveillance.

[18] Soria-Segarra C, Soria-Segarra C, Catagua-González A, Gutiérrez-Fernández J (2020). Carbapenemase producing Enterobacteriaceae in intensive care units in Ecuador: results from a multicenter study. J Infect Public Health.

[19] Khan AU, Maryam L, Zarrilli R (2017). Structure, Genetics and Worldwide Spread of New Delhi Metallo- β -lactamase ( NDM ): a threat to public health. BMC Microbiol.

[20] Haji SH, Aka STH, Ali FA (2021). Prevalence and characterisation of carbapenemase encoding genes in multidrug-resistant Gram-negative bacilli. PLoS One.

[21] Escandón-Vargas K, Reyes S, Gutiérrez S, Villegas MV (2017). The epidemiology of carbapenemases in Latin America and the Caribbean. Expert Rev Anti Infect Ther.

[22] Hansen GT (2021). Continuous evolution: perspective on the epidemiology of Carbapenemase resistance among enterobacterales and other gram-negative bacteria. Infect Dis Ther.

[23] Ecuador IN de I y SP del. Vigilancia de BLEE y Carbapenemasas en Ecuador período 2018–2021. Available from: http://www.investigacionsalud.gob.ec/webs/ram/wp-content/uploads/2022/11/2do-Boletin-CRN-Resistencia-Antimicrobiana.pdf. [cited 2023 Sep 20].

[24] Marquez-Ortiz RA, Haggerty L, Olarte N, Duarte C, Garza-Ramos U, Silva-Sanchez J (2017). Genomic epidemiology of NDM-1-encoding plasmids in latin American clinical isolates reveals insights into the evolution of multidrug resistance. Genome Biol Evol.

[25] Findlay J, Perreten V, Poirel L, Nordmann P (2022). Molecular analysis of OXA-48-producing Escherichia coli in Switzerland from 2019 to 2020. Eur J Clin Microbiol Infect Dis.

[26] Neidhöfer C, Buechler C, Neidhöfer G, Bierbaum G, Hannet I, Hoerauf A (2021). Global distribution patterns of Carbapenemase-encoding bacteria in a new light: clues on a role for ethnicity. Front Cell Infect Microbiol.

[27] van Duin D, Doi Y (2017). The global epidemiology of Carbapenemase-producing enterobacteriaceae. Virulence.

[28] Organizacion Panamericana de la Salud. Emergencia e incremento de nuevas combinaciones de carbapenemasas en Enterobacterales en Latinoamérica y el Caribe. 2021. Available from: https://www.paho.org/es/documentos/alerta-epidemiologica-emergencia-e-incremento-nuevas-combinacionescarbapenemasas. [cited 2023 Sep 20].

[29] Organización Panamericana de la Salud. Emergencia e incremento de nuevas combinaciones de carbapenemasas en Enterobacterales en Latinoamérica y el Caribe. Emerg E Incremento Nuevas Comb Carbapenemasas En Enterobacterales En Latinoam El Caribe. 2021;(Vim):2–8.

[30] Oliveira R, Castro J, Silva S, Oliveira H, Saavedra MJ, Azevedo NF (2022). Exploring the antibiotic resistance profile of clinical Klebsiella pneumoniae Isolates in Portugal. Antibiotics.

[31] González Rocha G, Vera Leiva A, Barría Loaiza C, Carrasco Anabalón S, Lima C, Aguayo Reyes A (2017). Infectología al Día KPC: Klebsiella pneumoniae carbapenemasa, principal carbapenemasa en enterobacterias KPC. Infectol Al Día.

[32] Malek A, McGlynn K, Taffner S, Fine L, Tesini B, Wang J (2019). Next-generation-sequencing-based hospital outbreak investigation yields insight into Klebsiella aerogenes population structure and determinants of Carbapenem resistance and pathogenicity. Antimicrob Agents Chemother.

[33] El-Mahallawy HA, El Swify M, Abdul Hak A, Zafer MM (2022). Increasing trends of colistin resistance in patients at high-risk of carbapenem-resistant Enterobacteriaceae. Ann Med.

[34] Zarate M, Barrantes D, Cuicapuza D, Velasquez J, Fernández N, Salvatierra G (2021). Frequency of colistin resistance in Pseudomonas aeruginosa: first report from Peru. Rev Peru Med Exp Salud Pública.

[35] Garza-Ramos U, Silva-Sánchez J, López-Jácome LE, Hernández-Durán M, Colín-Castro CA, Sánchez-Pérez A (2023). Carbapenemase-encoding genes and colistin resistance in gram-negative bacteria during the COVID-19 pandemic in Mexico: results from the Invifar network. Microb Drug Resist.

[36] Ortega-Paredes D, Barba P, Zurita J (2016). Colistin-resistant *Escherichia coli* clinical isolate harbouring the *mcr-1* gene in Ecuador. Epidemiol Infect.

[37] Calero-CÃ W (2023). Evolution and dissemination of mobile colistin resistance genes: limitations and challenges in Latin American countries. Lancet.

[38] Mendes Oliveira VR, Paiva MC, Lima WG (2019). Plasmid-mediated colistin resistance in Latin America and Caribbean: a systematic review. Travel Med Infect Dis.

[39] Daza-Cardona EA, Buenhombre J, Fontenelle RO dos S, Barbosa FCB (2022). mcr-mediated colistin resistance in South America, a One Health approach: a review. Rev Res Med Microbiol.

[40] Findlay J, Poirel L, Juhas M, Nordmann P (2021). KPC-mediated resistance to ceftazidime-avibactam and collateral effects in Klebsiella pneumoniae. Antimicrob Agents Chemother.

[41] Cui X, Shan B, Zhang X, Qu F, Jia W, Huang B (2020). Reduced Ceftazidime-Avibactam Susceptibility in KPC-Producing Klebsiella pneumoniae from patients without ceftazidime-avibactam use history – a multicenter study in China. Front Microbiol.

[42] Wozniak A, Paillavil B, Legarraga P, Zumarán C, Prado S, García P (2019). Evaluation of a rapid immunochromatographic test for detection of KPC in clinical isolates of Enterobacteriaceae and Pseudomonas species. Diagn Microbiol Infect Dis.

[43] Schäfer E, Malecki M, Tellez-Castillo CJ, Pfennigwerth N, Marlinghaus L, Higgins PG (2019). Molecular surveillance of carbapenemase-producing Pseudomonas aeruginosa at three medical centres in Cologne, Germany. Antimicrob Resist Infect Control.

[44] Gallagher JC, Satlin MJ, Elabor A, Saraiya N, McCreary EK, Molnar E (2018). Ceftolozane-tazobactam for the treatment of multidrug-resistant pseudomonas aeruginosa infections: a multicenter study. Open Forum Infect Dis.

[45] Ajila Acuña A, Satán Salazar C, Villavicencio F, Villacís JE. Determinación de la actividad de ceftolozane/tazobactam en cepas de. Rev Científica INSPILIP. 2022;6(1):9–18.

[46] Liu C, Chen K, Wu Y, Huang L, Fang Y, Lu J (2022). Epidemiological and genetic characteristics of clinical carbapenem-resistant Acinetobacter baumannii strains collected countrywide from hospital intensive care units ( ICUs ) in China. Emerg Microbes Infect.

[47] McKay SL, Vlachos N, Daniels JB, Albrecht VS, Stevens VA, Rasheed JK (2022). Molecular Epidemiology of Carbapenem-Resistant Acinetobacter baumannii in the United States, 2013–2017. Microb Drug Resist.

[48] Nuñez Quezada T, Rodríguez CH, Castro Cañarte G, Nastro M, Balderrama Yarhui N, Dabos L, Acosta Mosquera Y, Plaza Moreira N, Famiglietti A. Outbreak of blaOXA-72-producing Acinetobacter baumannii in South America. J Chemother. 2017;29(5):321–4. 10.1080/1120009X.2016.1158936. 10.1080/1120009X.2016.1158936 27077936

[49] Rodríguez CH, Balderrama Yarhui N, Nastro M, Nuñez Quezada T, Castro Cañarte G, Magne Ventura R (2016). Molecular epidemiology of carbapenem-resistant Acinetobacter baumannii in South America. J Med Microbiol.

[50] Chen Y, Yang Y, Liu L, Qiu G, Han X, Tian S (2018). High prevalence and clonal dissemination of OXA-72-producing Acinetobacter baumannii in a Chinese hospital: a cross sectional study. BMC Infect Dis.

[51] Thirapanmethee K, Srisiri-a-nun T, Houngsaitong J, Montakantikul P, Khuntayaporn P, Chomnawang MT (2020). xcPrevalence of OXA-Type β-lactamase genes among Carbapenem-resistant acinetobacter baumannii clinical isolates in Thailand. BMC Infect Dis.

[52] Gutiérrez S, Correa A, Hernández-Gómez C, De La Cadena E, Pallares C, Villegas MV (2019). Detection of carbapenemase-producing Pseudomonas aeruginosa: evaluation of the carbapenem inactivation method (CIM). Enfermedades Infecc Microbiol Clin Engl Ed.

