# Supplementary material for: Molecular epidemiology of carbapenem-resistant gram-negative bacilli in Ecuador
Source: BMC Infect Dis. 2024 Apr 6;24:378. doi: 10.1186/s12879-024-09248-6 (PMC10998298; doi:10.1186/s12879-024-09248-6)
Supplement: Supplementary file 1 — Supplementary Material 1. [file 12879_2024_9248_MOESM1_ESM.docx]

**Supplementary table No. 1. Minimum inhibitory concentration values**

|  | **MIC *µ*g/ml** | | | | | |  |  |  |  |  |  |
| --- | --- | --- | --- | --- | --- | --- | --- | --- | --- | --- | --- | --- |
| **AMIKACIN** | **<=2** | **4** | **8** | **16** | **32** | **>=64** |  | No isolates tested |  |  |  |  |
| ***K. pneumoniae*** | 1 | 20 |  |  | 14 | 10 |  | 45 |  |  |  |  |
| ***K. aerogenes*** | 2 |  |  |  |  | 8 |  | 10 |  |  |  |  |
| ***E. cloacae*** | 2 |  |  |  |  | 1 |  | 3 |  |  |  |  |
| ***E. coli*** | 1 |  |  |  |  | 1 |  | 2 |  |  |  |  |
| ***P. aeruginosa*** |  | 4 |  | 2 | 3 | 2 |  | 11 |  |  |  |  |
| ***A. baumannii* complex** |  |  |  |  | 3 | 18 |  | 21 |  |  |  |  |
|  |  |  |  |  |  |  |  |  |  |  |  |  |
| **GENTAMICIN** | **<=2** | **4** | **8** | **16** | **32** | **>=64** |  |  |  |  |  |  |
| ***K. pneumoniae*** | 7 | 1 | 2 | 1 |  | 34 |  | 45 |  |  |  |  |
| ***K. aerogenes*** | 2 |  |  |  |  | 8 |  | 10 |  |  |  |  |
| ***E. cloacae*** |  |  |  |  |  | 3 |  | 3 |  |  |  |  |
| ***E. coli*** | 1 |  |  |  |  | 1 |  | 2 |  |  |  |  |
| ***P. aeruginosa*** | 1 | 2 | 4 |  | 4 |  |  | 11 |  |  |  |  |
| ***A. baumannii* complex** |  |  |  |  |  | 21 |  | 21 |  |  |  |  |
|  |  |  |  |  |  |  |  |  |  |  |  |  |
| **CIPROFLOXACIN** | **<=0.25** | **0.5** | **1** | **2** | **>=4** |  |  |  |  |  |  |  |
| ***K. pneumoniae*** | 6 |  | 2 |  | 37 |  |  | 45 |  |  |  |  |
| ***K. aerogenes*** | 2 |  |  |  | 8 |  |  | 10 |  |  |  |  |
| ***E. cloacae*** |  |  |  |  | 3 |  |  | 3 |  |  |  |  |
| ***E. coli*** |  | 1 |  |  | 1 |  |  | 2 |  |  |  |  |
| ***P. aeruginosa*** |  | 2 | 1 |  | 8 |  |  | 11 |  |  |  |  |
| ***A. baumannii* complex** |  |  |  |  | 21 |  |  | 21 |  |  |  |  |
|  |  |  |  |  |  |  |  |  |  |  |  |  |
| **TIGECICLINA** | **<=0,5** | **1** | **2** | **4** | **>=8** |  |  |  |  |  |  |  |
| ***K. pneumoniae*** | 27 | 7 | 9 | 2 |  |  |  | 45 |  |  |  |  |
| ***K. aerogenes*** | 10 |  |  |  |  |  |  | 10 |  |  |  |  |
| ***E. cloacae*** | 2 |  |  |  | 1 |  |  | 3 |  |  |  |  |
| ***E. coli*** | 2 |  |  |  |  |  |  | 2 |  |  |  |  |
|  |  |  |  |  |  |  |  |  |  |  |  |  |
| **TRIMETHROPIM/SULFAMETHOXAZOL** | **<=1/19** | **2/38** | **4/76** | **>=16/304** |  |  |  |  |  |  |  |  |
| ***K. pneumoniae*** | 4 |  | 41 |  |  |  |  | 45 |  |  |  |  |
| ***K. aerogenes*** |  |  | 10 |  |  |  |  | 10 |  |  |  |  |
| ***E. cloacae*** |  |  | 3 |  |  |  |  | 3 |  |  |  |  |
| ***E. coli*** | 1 |  | 1 |  |  |  |  | 2 |  |  |  |  |
| ***A. baumannii* complex** |  |  |  | 21 |  |  |  | 21 |  |  |  |  |
|  |  |  |  |  |  |  |  |  |  |  |  |  |
| **CEFTAZIDIME** | **2** | **4** | **8** | **16** | **32** | **>=64** |  |  |  |  |  |  |
| ***K. pneumoniae*** |  |  |  |  |  | 45 |  | 45 |  |  |  |  |
| ***K. aerogenes*** |  |  |  |  |  | 10 |  | 10 |  |  |  |  |
| ***E. cloacae*** |  |  |  |  |  | 3 |  | 3 |  |  |  |  |
| ***E. coli*** |  |  |  |  |  | 2 |  | 2 |  |  |  |  |
| ***P. aeruginosa*** |  | 1 | 1 |  | 1 | 8 |  | 11 |  |  |  |  |
| ***A. baumannii* complex** |  |  | 4 | 2 |  | 18 |  | 24 |  |  |  |  |
|  |  |  |  |  |  |  |  |  |  |  |  |  |
| **CEFEPIME** | **<=1** | **2** | **4** | **8** | **16** | **>=32** |  |  |  |  |  |  |
| ***K. pneumoniae*** |  |  |  |  |  | 45 |  | 45 |  |  |  |  |
| ***K. aerogenes*** |  |  |  |  |  | 10 |  | 10 |  |  |  |  |
| ***E. cloacae*** |  |  |  |  |  | 3 |  | 3 |  |  |  |  |
| ***E. coli*** | 1 |  |  |  |  | 1 |  | 2 |  |  |  |  |
| ***P. aeruginosa*** |  |  |  | 1 |  | 10 |  | 11 |  |  |  |  |
| ***A. baumannii* complex** |  |  |  |  | 14 | 10 |  | 24 |  |  |  |  |
|  |  |  |  |  |  |  |  |  |  |  |  |  |
| **IMIPENEM** | **<=0.25** | **1** | **2** | **4** | **8** | **>=16** |  |  |  |  |  |  |
| ***K. pneumoniae*** |  |  |  |  |  | 45 |  | 45 |  |  |  |  |
| ***K. aerogenes*** |  |  |  |  |  | 10 |  | 10 |  |  |  |  |
| ***E. cloacae*** |  |  |  |  |  | 3 |  | 3 |  |  |  |  |
| ***E. coli*** |  | 1 | 1 |  |  |  |  | 2 |  |  |  |  |
| ***P. aeruginosa*** |  |  |  |  |  | 11 |  | 11 |  |  |  |  |
| ***A. baumannii* complex** |  |  |  |  |  | 24 |  | 24 |  |  |  |  |
|  |  |  |  |  |  |  |  |  |  |  |  |  |
| **MEROPENEM** | **<=0.25** | **1** | **2** | **4** | **8** | **>=16** |  |  |  |  |  |  |
| ***K. pneumoniae*** |  |  |  |  |  | 45 |  | 45 |  |  |  |  |
| ***K. aerogenes*** |  |  |  |  |  | 10 |  | 10 |  |  |  |  |
| ***E. cloacae*** |  |  |  |  |  | 3 |  | 3 |  |  |  |  |
| ***E. coli*** |  | 1 | 1 |  |  |  |  | 2 |  |  |  |  |
| ***P. aeruginosa*** |  |  |  |  |  | 11 |  | 11 |  |  |  |  |
| ***A. baumannii* complex** |  |  |  |  |  | 24 |  | 24 |  |  |  |  |
|  |  |  |  |  |  |  |  |  |  |  |  |  |
| **PIPERACILLIN/TAZOBACTAM** | **16/4** | **32/4** | **64/4** | **>=128/4** |  |  |  |  |  |  |  |  |
| ***K. pneumoniae*** |  |  |  | 45 |  |  |  | 45 |  |  |  |  |
| ***K. aerogenes*** |  |  |  | 10 |  |  |  | 10 |  |  |  |  |
| ***E. cloacae*** |  |  |  | 3 |  |  |  | 3 |  |  |  |  |
| ***E. coli*** |  |  |  | 2 |  |  |  | 2 |  |  |  |  |
| ***P. aeruginosa*** |  |  |  | 11 |  |  |  | 11 |  |  |  |  |
| ***A. baumannii* complex** |  |  |  | 24 |  |  |  | 24 |  |  |  |  |
|  |  |  |  |  |  |  |  |  |  |  |  |  |
| **CEFTAZIDIME/AVIBACTAM** | **<=0.12/4** |  | **3/4** | **4/4** | **8/4** | **16/4** |  |  |  |  |  |  |
| ***K. pneumoniae*** | 37 |  | 2 | 1 |  |  |  | 40 |  |  |  |  |
| ***K. aerogenes*** | 9 |  |  |  |  |  |  | 9 |  |  |  |  |
| ***E. cloacae*** | 2 |  |  |  |  |  |  | 2 |  |  |  |  |
|  |  |  |  |  |  |  |  |  |  |  |  |  |
| **COLISTIN** | **<=1** | **1** | **2** | **4** | **>=8** |  |  |  |  |  |  |  |
| ***K. pneumoniae*** |  | 39 |  |  | 6 |  |  | 45 |  |  |  |  |
| ***K. aerogenes*** |  | 2 |  |  | 8 |  |  | 10 |  |  |  |  |
| ***E. cloacae*** |  | 3 |  |  |  |  |  | 3 |  |  |  |  |
| ***E. coli*** |  | 2 |  |  |  |  |  | 2 |  |  |  |  |
| ***P. aeruginosa*** |  | 6 | 1 | 4 |  |  |  | 11 |  |  |  |  |
| ***A. baumannii* complex** |  | 18 | 1 | 1 |  |  |  | 20 |  |  |  |  |
|  |  |  |  |  |  |  |  |  |  |  |  |  |
|  |  |  |  |  |  |  |  |  |  |  |  |  |
|  |  |  |  |  |  |  |  |  |  |  |  |  |
| **AMPICILLIN/SULBACTAM** | **8/4** | **16/8** | **>=32/16** |  |  |  |  |  |  |  |  |  |
| ***A. baumannii* complex** | 2 | 3 | 19 |  |  |  |  | 24 |  |  |  |  |
|  |  |  |  |  |  |  |  |  |  |  |  |  |
| **CEFTOLOZANE/TAZOBACTAM** | **1.5/4** | **4/4** | **8/4** | **>=16/4** |  |  |  |  |  |  |  |  |
| ***P. aeruginosa*** | 2 | 1 |  |  |  |  |  | 3 |  |  |  |  |
|  |  |  |  |  |  |  |  |  |  |  |  |  |
| **MEROPENEM/VABORBACTAM** | **0.025/8** | **0.032/8** | **0.047/8** | **0.064/8** | **0.25/8** | **0.32/8** | **0.47/8** | **0.5/8** | **0.94/8** | **1/8** | **3/8** | **24/8** |
| ***K. pneumoniae*** | 1 | 2 | 4 | 4 | 1 | 3 | 5 | 1 | 1 | 1 |  |  |
| ***K. aerogenes*** |  |  |  |  |  |  |  | 1 |  |  |  |  |
| ***E. cloacae*** |  | 1 |  |  |  |  |  |  |  |  |  |  |
| ***E. coli*** |  |  |  |  |  |  |  |  |  |  |  |  |
| ***P. aeruginosa**** |  |  |  |  |  |  |  | 1 |  | 1 | 1 |  |

* EUCAST breakpoints

| Susceptible |
| --- |
| Intermediate |
| Resistant |
