# Supplementary material for: Molecular epidemiology of carbapenem-resistant gram-negative bacilli in Ecuador
Source: BMC Infect Dis. 2024 Apr 6;24:378. doi: 10.1186/s12879-024-09248-6 (PMC10998298; doi:10.1186/s12879-024-09248-6)
Supplement: Supplementary file 3 — Supplementary Material 3. [file 12879_2024_9248_MOESM3_ESM.pdf]

Supplementary figure No. 2. Dendrogram results of *bla*$_{KPC}$-positive *K. pneumoniae*

Genetic distance

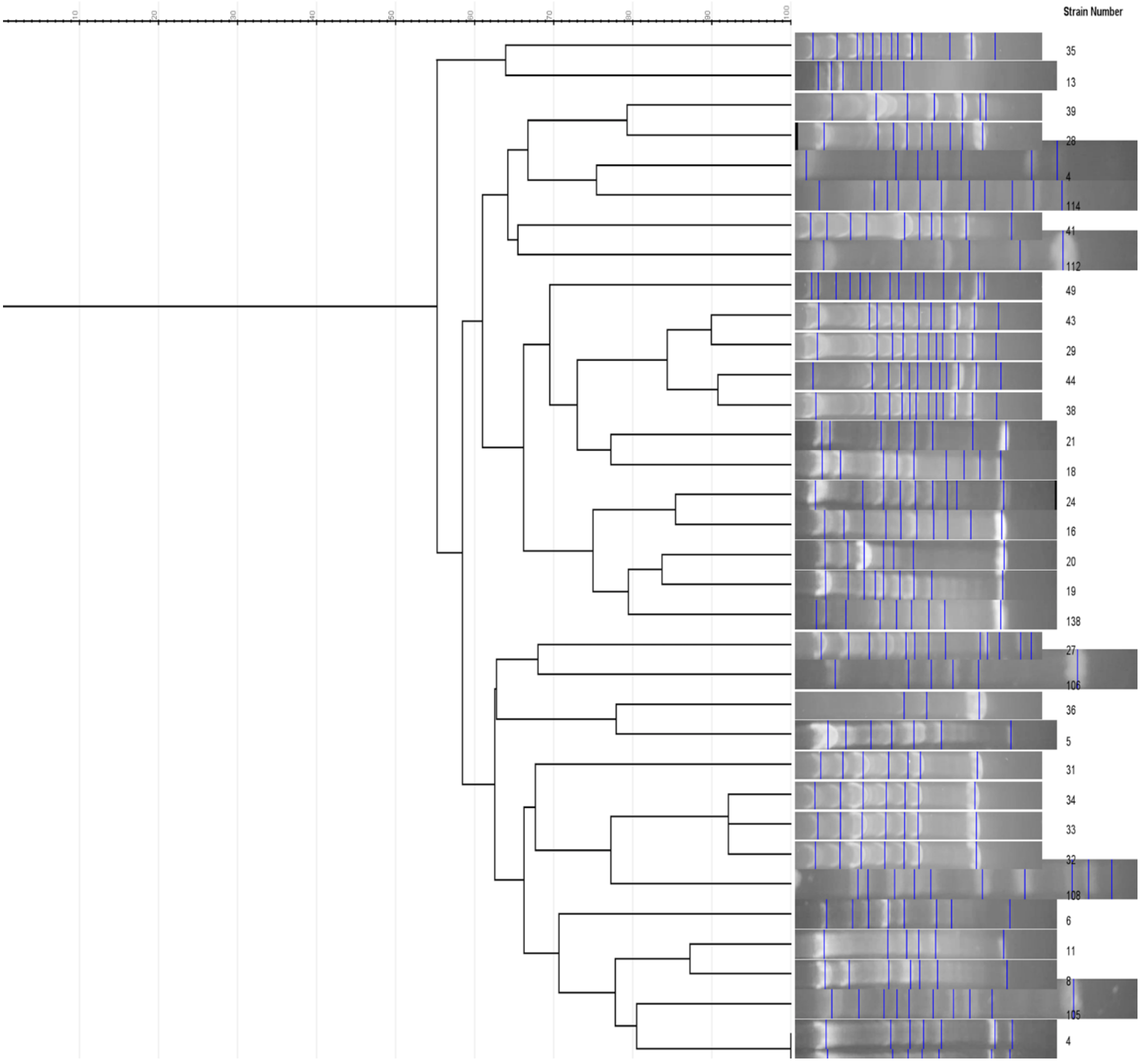

ERIC-PCR fingerprinting of *K. pneumoniae.* Thirty-five isolates studied
