# Supplementary material for: Molecular epidemiology of carbapenem-resistant gram-negative bacilli in Ecuador
Source: BMC Infect Dis. 2024 Apr 6;24:378. doi: 10.1186/s12879-024-09248-6 (PMC10998298; doi:10.1186/s12879-024-09248-6)
Supplement: Supplementary file 4 — Supplementary Material 4. [file 12879_2024_9248_MOESM4_ESM.pdf]

Supplementary figure No. 3. Dendrogram results of *bla*$_{OXA\ 24/40}$-positive *A. baumannii* complex

Genetic distance

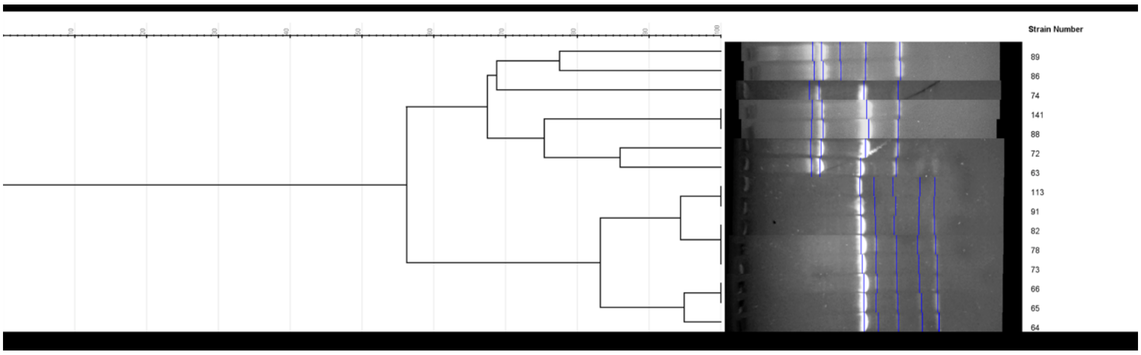

ERIC-PCR fingerprinting of *A. baumannii* complex
