# Supplementary material for: Molecular epidemiology of carbapenem-resistant gram-negative bacilli in Ecuador
Source: BMC Infect Dis. 2024 Apr 6;24:378. doi: 10.1186/s12879-024-09248-6 (PMC10998298; doi:10.1186/s12879-024-09248-6)
Supplement: Supplementary file 5 — Supplementary Material 5. [file 12879_2024_9248_MOESM5_ESM.pdf]

Supplementary figure No. 4. Dendrogram results of *bla*$_{KPC}$-positive *K. aerogenes.*

Genetic distance

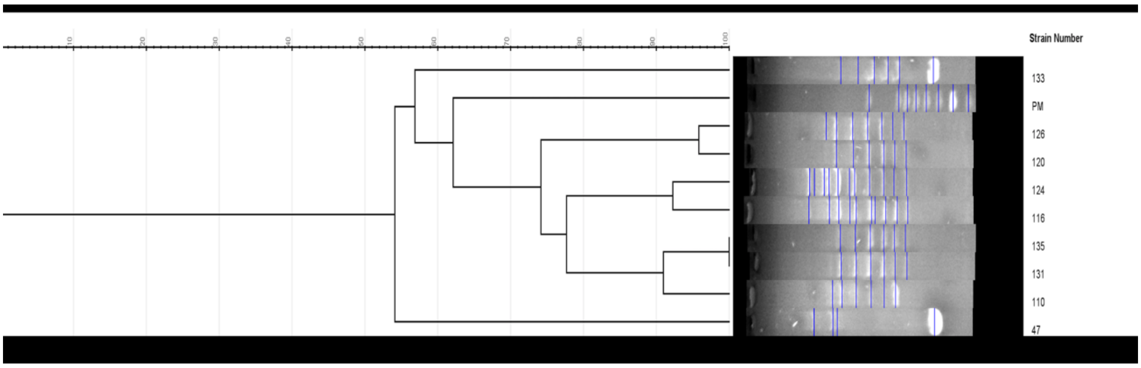

ERIC-PCR fingerprinting of K.*aerogenes*
